# Breaking the Cycle of Echinococcosis: A Mathematical Modeling Approach

**DOI:** 10.3390/tropicalmed10040101

**Published:** 2025-04-09

**Authors:** Richard Lagos, Juan Pablo Gutiérrez-Jara, Beatriz Cancino-Faure, Leidy Yissedt Lara-Díaz, Ignacio Barradas, Andrei González-Galeano

**Affiliations:** 1 Programa de Doctorado en Modelamiento Matemático Aplicado, Facultad de Ciencias Básicas, Universidad Católica del Maule, Talca 3480112, Chile; 2 Departamento de Matemática y Física, Facultad de Ciencias, Universidad de Magallanes, Punta Arenas P.O. Box 113-D, Chile; 3 Centro de Investigación de Estudios Avanzados del Maule (CIEAM), Vicerrectoría de Investigación y Postgrado, Universidad Católica del Maule, Talca 3480112, Chile; 4 Laboratorio de Microbiología y Parasitología, Departamento de Ciencias Preclínicas, Facultad de Medicina, Universidad Católica del Maule, Talca 3480112, Chile; bcancino@ucm.cl; 5 Departamento de Matemática, Física y Estadística, Facultad de Ciencias Básicas, Universidad Católica del Maule, Talca 3480112, Chile; lelara@ucm.cl; 6 Centro de Investigación en Matemáticas, Guanajuato 36023, Mexico; barradas@cimat.mx (I.B.); andrei.gonzalez@cimat.mx (A.G.-G.); 7 Departamento de Matemáticas, Facultad de Ciencias, Universidad El Bosque, Bogotá 111321, Colombia

**Keywords:** *Echinococcus granulosus*, compartmental model, hydatidosis, neglected tropical disease, zoonosis

## Abstract

This study presents a mathematical model of the transmission and spread of the *Echinococcus granulosus* parasite. The model incorporates host mobility, laws governing the dynamics of Echinococcosis transmission between hosts, and control and prevention measures. The basic reproductive number of the proposed model is calculated, and a sensitivity analysis is performed to identify the parameters that most influence the dynamics of transmission and spread of the disease among its hosts. The study evaluates two control strategies—dog deworming and sheep vaccination—based on their respective target reproductive numbers. The impact of these control and prevention measures is investigated through numerical simulations, which reveal that the dog deworming strategy consistently reduces infections in humans. In contrast, the sheep vaccination strategy demonstrates a more favorable scenario for disease eradication in both hosts. In addition, simulations show a close relationship between the early detection of the disease and the recovery of the patient.

## 1. Introduction

Cystic echinococcosis (CE) is a zoonotic disease caused by the parasite *Echinococcus granulosus* [1]. This parasite requires two mammalian hosts to complete its life cycle: the adult stage develops in the intestines of dogs, while the larval stage forms cysts primarily in the organs of sheep [2]. The domestic transmission cycle begins when dogs consume the organs of infected sheep, releasing *E. granulosus* eggs into the environment through their feces. These eggs can contaminate crops and water, which sheep may ingest during grazing, perpetuating the cycle. The disease predominantly occurs in rural areas, where sheep farming is the main productive activity, due to the common practice of feeding dogs with offal from infected sheep. However, as rural populations migrate to peri-urban areas, where small flocks of sheep are maintained and the practice of feeding dogs with offal continues, the geographical distribution has expanded, increasing its potential public health impact [3,4]. The informal slaughter and marketing of sheep meat, frequently observed at urban fairs [5], is another factor contributing to the spread of CE.

Humans contract the infection upon ingesting viable *E. granulosus* eggs [6,7]. In most cases, the asymptomatic phase of the disease can last over 10 years, depending on the affected organ, until hydatid cysts grow large enough to cause clinical symptoms [6,8]. This disease imposes a significant clinical and socioeconomic burden, particularly in adults, as it may lead to disability, prolonged work absenteeism, and substantial healthcare costs associated with hospitalization and surgery [9].

The World Health Organization (WHO) has classified CE as a neglected tropical disease. The Pan American Health Organization (PAHO) reported 54,527 human cases in South America (Argentina, Brazil, Chile, Paraguay, Peru, and Uruguay) between 2009 and 2021 [10]. Successful control measures have been implemented in Iceland, Tasmania, and New Zealand to eradicate CE in humans and animals [11]. As an initiative of the PAHO/WHO and in collaboration with the International Association of Hydatidology, the Regional Program for the Elimination of Cystic Echinococcosis/Hydatidosis (2020–2029) has been created. The program’s action plan includes strengthening human and animal surveillance and diagnosis, human case management, animal host intervention, and community education. However, *E. vogeli*, *E. oligarthrus*, and *E. multilocularis* are also present in South, Central, and North America, corresponding to the etiologic agents of polycystic, unicystic, and alveolar echinococcosis, respectively, and differing in their epidemiology concerning cystic echinococcosis/hydatidosis [12].

Mathematical models have been widely applied to study *E. granulosus’* transmission dynamics and evaluate control strategies. In a study by Wu et al. [13], the role of human intervention through dog deworming and environmental disinfection was analyzed. Similarly, Birhan et al. [14] focused on vaccination and environmental disinfection.

The role of human mobility in the spread of infectious diseases has been the subject of numerous studies in the scientific literature. Among them, the study by Gutiérrez-Jara et al. [15] examined the impact of human mobility on the transmission of hantaviruses. The authors found that human mobility plays a crucial role in the emergence and spread of zoonotic diseases. Khan et al. [16] employed data-driven correlation analysis to investigate the potential for international air travel to facilitate global epidemics, highlighting the potential for such modes of transportation to contribute to the spread of infectious diseases on a global scale.

The mathematical models for the transmission dynamics of the infection proposed in the studies [13,14,15] are of the compartmental type, in which the incidence of the disease is represented in terms of a constant transmission rate. In their studies, Córdova-Lepe and Vogt-Geisse [17] and Córdova-Lepe and Gutiérrez-Jara [18] present SEIR- and SIR-type mathematical models for the transmission of SARS-CoV-2. These models incorporate a dynamic law for the transmission rate of the disease that allows for the consideration of temporal variability in relation to the behavior of the human population.

CE is a controllable zoonosis, as evidenced by the efficacy of the periodic deworming of dogs and vaccination of sheep [11]. Despite these measures, it persists as an endemic disease in numerous countries, particularly in South America. The PAHO and the WHO have classified it as a neglected zoonosis affecting underserved populations. According to these organizations, the eradication of the disease is contingent upon the availability of resources for each control measure and their sustained application over time. From a theoretical perspective, it would be beneficial to have a tool to evaluate the effectiveness of control measures a priori in order to make informed decisions and plan the eradication of the disease with the available resources.

The objective of this study was to evaluate the impact of control and prevention measures on the domestic transmission cycle of CE in peri-urban, urban, and rural areas. To this end, a compartmental model was developed that incorporates dog deworming, sheep vaccination, and preventive exams in the human population.

## 2. Materials and Methods

### 2.1. Compartmental Model

We propose a strategic deterministic mathematical model for the domestic life cycle of the transmission, spread, and control dynamics of *E. granulosus*, involving the dog (*D*) and the sheep (*O*). In the event of the accidental introduction of an adult (*A*) or juvenile (*J*) into the transmission cycle of the disease, the average latency period before the onset of symptoms is estimated to be ten years, based on the observation that the primoinfection as an adult typically occurs in rural locations where dogs are frequently fed animal viscera containing hydatid cysts, thereby initiating the life cycle of the parasite.

In our mathematical model, we divide each of the total host populations into mutually exclusive groups (compartments) according to their epidemiological states.

The population of peri-urban (*P*), urban (*U*), and rural (*R*) dogs was classified into three epidemiological states: susceptible, infected, and treated. Dogs that were free of disease were classified as susceptible (*s*), while those that had contracted the disease and were infectious were designated as infected (*i*). Finally, those that had undergone deworming treatment were classified as treated (*T*). The sizes of these populations were designated as $D_{P}^{s},\;D_{U}^{s},\;D_{R}^{s},$ $D_{P}^{i},\;D_{U}^{i},\;D_{R}^{i},$ $D_{P}^{T},\;D_{U}^{T},$ and $D_{R}^{T},$ respectively. The total population size of the dog population, $N^{D},$ was the sum of the sizes of these states: $N^{D}=D_{P}^{s}+D_{P}^{i}+D_{P}^{T}+D_{U}^{s}+D_{U}^{i}+D_{U}^{T}+D_{R}^{s}+D_{R}^{i}+D_{R}^{T}$.

The population of peri-urban (*P*) and rural (*R*) sheep was divided into three epidemiological states: susceptible, infected, and vaccinated. Healthy sheep were classified as susceptible (*s*); sheep with hydatid cysts were classified as infected (*i*); and sheep undergoing vaccination were classified as vaccinated (*v*). The size of these populations is denoted by $O_{P}^{s},\;O_{R}^{s},$ $O_{P}^{i},\;O_{R}^{i},$ $O_{P}^{v},$ and $O_{R}^{v},$ respectively. The total size of the sheep population $N^{O}$ is the sum of the sizes of these states: $N^{O}=O_{P}^{s}+O_{P}^{i}+O_{P}^{v}+O_{R}^{s}+O_{R}^{i}+O_{R}^{v}$.

The population of peri-urban, urban, and rural juveniles and adults was divided into four and five epidemiological states, respectively. Healthy humans were classified as susceptible (*s*); those with disease who were not detected by early preventive exams were classified as undetected (*u*); humans with the disease but with no clinical signs or symptoms of the disease who were treated with pharmacological agents were classified as treated level 1 (L1); humans with the disease and with clinical signs and symptoms who underwent surgery were classified as treated level 2 (L2); and humans who had successfully survived treatment were classified as recovered (*r*). The size of each of these populations is denoted by $J_{P}^{s},\;J_{P}^{u},\;J_{P}^{L1},\;J_{P}^{r},\;J_{U}^{s},\;J_{U}^{u},\;J_{U}^{L1},\;J_{U}^{r},\;J_{R}^{s},\;J_{R}^{u},\;J_{R}^{L1},\;J_{R}^{r},\;A_{P}^{s},\;A_{P}^{u},\;A_{P}^{L1},\;A_{P}^{L2},\;A_{P}^{r},\;A_{U}^{s},\;A_{U}^{u},\;A_{U}^{L1},\;A_{U}^{L2},\;A_{U}^{r},\;A_{R}^{s},$ $A_{R}^{u},\;A_{R}^{L1},\;A_{R}^{L2},\;A_{R}^{r}$. The total size of these human populations $N^{J}$ and $N^{A}$ corresponds to the sum of the sizes of these four and six epidemic states, respectively: $N_{J}=J_{P}^{s}+J_{P}^{u}+J_{P}^{L1}+J_{P}^{r}+J_{U}^{s}+J_{U}^{u}+J_{U}^{L1}+J_{U}^{r}+J_{R}^{s}+J_{R}^{u}+J_{R}^{L1}+J_{R}^{r},$ and $N_{A}=A_{P}^{s}+A_{P}^{u}+A_{P}^{L1}+A_{P}^{L2}+A_{P}^{r}+A_{U}^{s}+A_{U}^{u}+A_{U}^{L1}+A_{U}^{L2}+A_{U}^{r}+A_{R}^{s}+A_{R}^{u}+A_{R}^{L1}+A_{R}^{L2}+A_{R}^{r}$.

To incorporate the demographic factor regarding the populations of dogs, sheep, and humans, our model assumes that the birth rate of each population is proportional to its size ($\mu_{X}N^{X},\;X\in \left\{D,O,J,A\right\}$). Furthermore, it is assumed that the birth and death rates per capita are equal for each population, i.e., $\mu_{X}=d_{X},$ which implies that the total size of each population remains constant.

**Figure 1:**
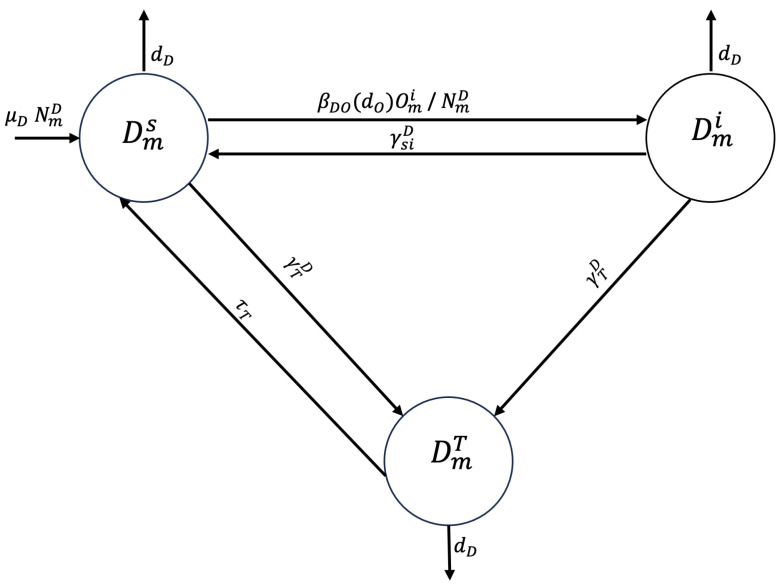
Flowchart for the dog population epidemiological stage compartment model.

A susceptible dog from zone $m,\;m\in \left\{P,U,R\right\},\;D_{m}^{s},$ may ingest viscera from infected sheep and become infected at transmission rate $\beta_{DO},$ entering the infected epidemiological state ($D_{m}^{i}$). Alternatively, the dog may receive deworming treatment at rate $\gamma_{T}^{D}$ and transition to the treated epidemiological state ($D_{m}^{T}$), or it may die a natural death at rate $d_{D}$. The rate at which an infected dog becomes susceptible again, depending on the half-life of the parasite *E. granulosus*, is represented by $\gamma_{si}^{D}$. The dog may be treated with deworming at rate $\gamma_{T}^{D}$ or may die a natural death. A dog treated with deworming can either die a natural death or become susceptible at rate $\tau_{T}$ (see Figure 1).

**Figure 2:**
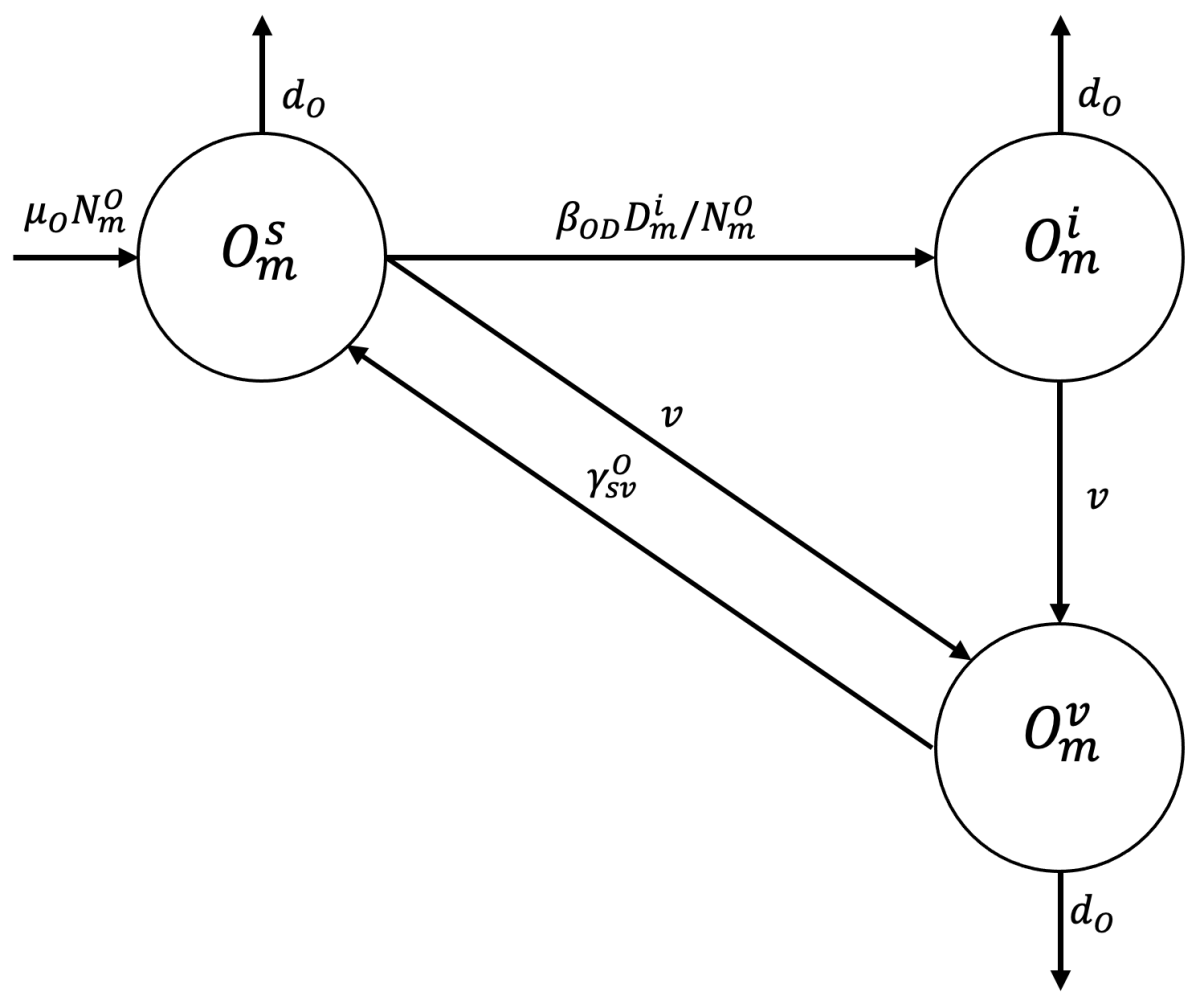
Flowchart for the sheep population epidemiological stage compartment model.

A susceptible sheep in zone $m,\;m\in \left\{P,U,R\right\},\;O_{m}^{s},$ may become infected by ingesting food or water contaminated with dog feces containing viable *E. granulosus* eggs at disease transmission rate $\beta_{OD},$ and enter the infected epidemiologic state $O_{m}^{i},$ or be vaccinated at vaccination rate *v* and enter state $O_{m}^{v},$ or die of natural causes at rate $d_{O}$. The sheep may become infected at rate $\beta_{OD}\delta_{v}$ and enter the infected state or die of natural causes (see Figure 2).

**Figure 3:**
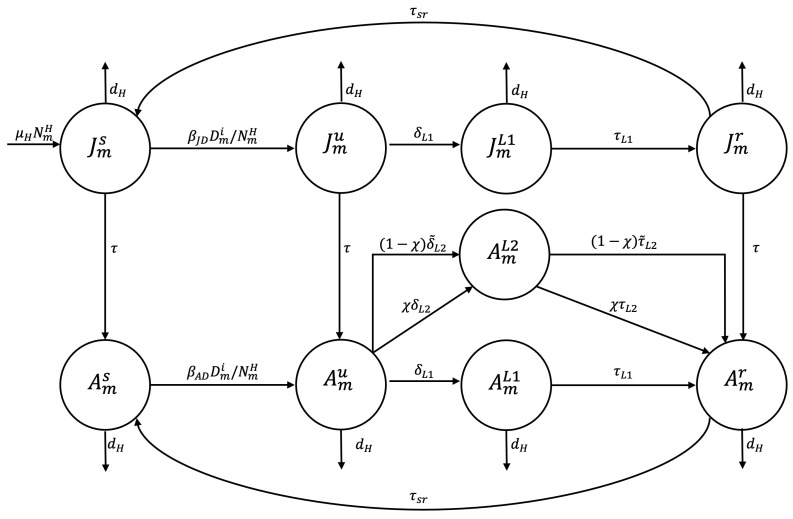
Flowchart for the human population epidemiological stage compartment model.

The infection of a susceptible adult or juvenile in zone m,m∈{P,U,R}, occurs when they come into contact with an infected dog or consume water or food contaminated with dog feces containing viable *E. granulosus* eggs. The disease transmission rate is $\beta_{AD}$ or $\beta_{JD},$ respectively, resulting in the individual entering the undetected infected epidemiological state ($A_{m}^{u}$ or $J_{m}^{u}$). A susceptible human may die of natural causes at a rate of $d_{H}$. A human in the undetected infected epidemiological state may undergo early preventive examination and be transferred to the treated exposed state to comply with drug treatment, at a detection rate of $\delta_{L1}$. Following the successful completion of this treatment at an average time $1/\tau_{L1},$ the subject transitions to the recovered state until they lose immunity and enter the susceptible state at a rate $\tau_{sr},$ or until they die of natural causes. An adult in the undetected infected state may undergo early preventive screening and transition to the treated infected state for surgical intervention at an infected detection rate of $\delta_{L2}$. Alternatively, they may die a natural death. Following the successful completion of surgery within an average time frame of $1/\tau_{L2},$ the patient transitions to the recovered state until immunity is lost, at which point they enter the susceptible state at rate $\tau_{sr}$. A juvenile in the susceptible, undetected infected, or recovered epidemiological state may reach the adult stage at rate τ and transition to the susceptible, undetected infected, or recovered adult epidemiological state, respectively (see Figure 3).

**Figure 4:**
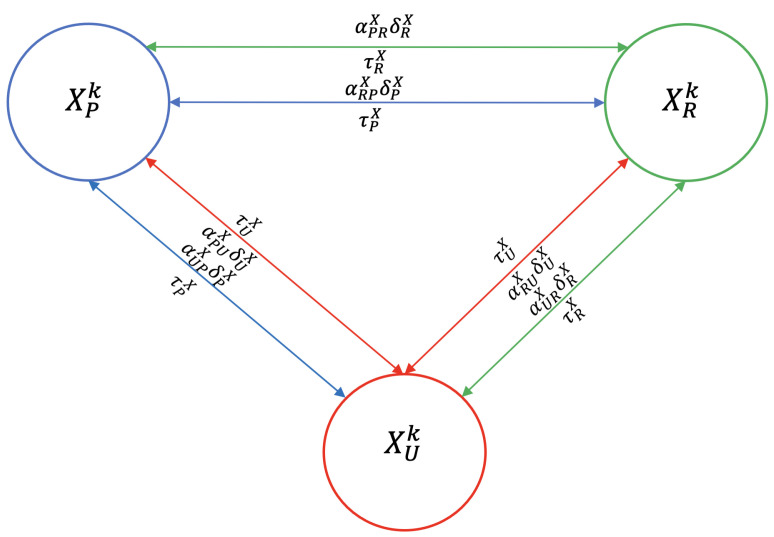
Flowchart of host mobility among peri-urban (blue), urban (red), and rural (green) areas, for host X. The direction of the arrow indicates the direction of host movement. A fraction (α) of the host population migrates to the other two zones at a rate of δ, where they remain for an average duration of 1/τ.

To model the mobility of hosts (dogs, sheep, and humans) between peri-urban, urban, and rural areas, we introduce mobility parameters. For simplicity, we denote by $X_{m}^{k}$ the size of the host population of zone *m* in epidemiological state k, where X∈{D,O,J,A}, m∈{P,U,R} and k∈{s,i,v,T,u,L1,L2,r}. Thus, $\delta_{m}^{X}$ represents the exit rate of host *X* from zone m, $\alpha_{nm}^{X}$ represents the fraction of hosts *X* from zone *n* that move to zone *m*, and $1/\tau_{m}^{X}$ represents the average time that a host *X* from zone *m* stays in another zone (see Figure 4). For example, $\alpha_{UR}^{D}$ is the fraction of dogs from the rural area that move to the urban area at rate $\delta_{R}^{D}$ and remain there for an average time of $1/\tau_{R}^{D}$.

The model parameters are summarized in Table 1 and Table 2.

### 2.2. Dynamics of Disease Transmission Rates

In our compartmental mathematical model detailed in Appendix A.1, we have utilized coefficients $\beta_{DO},\;\beta_{OD},\;\beta_{JD}$, and $\beta_{AD}$ to represent the incidence of disease in dogs, sheep, adults, and juveniles, respectively. In the field of mathematical epidemiology, these coefficients are known as transmission rates. We propose a dynamic for each of these transmission rates, wherein their numerical values decrease upon the implementation of disease control measures and revert to their initial values upon discontinuation of these measures.

The presence of *E. granulosus* in the environment is typically identified through a diagnosis of the parasite in dog feces. This detection facilitates the establishment of disease prevalence and transmission risk levels. The analysis of these results enables the definition of environmental infestation indices, which subsequently allow for the classification of different risk levels and the designation of control areas. The infestation index for area *m* (m∈{P,U,R}) is defined as the ratio of the number of positive samples to the total number of samples examined in that area: ${\tilde{\lambda }}_{m}=\lambda_{m}/\lambda_{T},$ where $\lambda_{T}=\lambda_{P}+\lambda_{U}+\lambda_{R}$ [10]. Another indicator to evaluate the effect of a CE control measure is the proportional mortality due to CE defined as the ratio of the number of hosts that died from echinococcosis per year to the total number of hosts that died per year. Thus, $\pi_{m}=\alpha_{O}/d_{O},$ where $\alpha_{s}$ is the per capita CE mortality rate and $d_{O}$ is the per capita sheep mortality rate, considering the proportional CE mortality of zone m,m∈{P,U,R}.

Thus, we use the differential equations

$${\dot{\beta }}_{DO}\left(t;m\right):=\left(\pi_{m}\left(1-\frac{\beta_{DO}^{m}\left(t\right)}{\beta_{DOmax}}\right)-\frac{O_{m}^{v}}{N_{m}^{O}}\right)\beta_{DO}^{m}\left(t\right);\;\beta_{DO}\left(0\right)=\beta_{DO}^{0},$$

$${\dot{\beta }}_{OD}\left(t;m\right):=\left({\tilde{\lambda }}_{m}\left(1-\frac{\beta_{OD}^{m}\left(t\right)}{\beta_{ODmax}}\right)-\frac{D_{m}^{T}}{N_{m}^{D}}\right)\beta_{OD}^{m}\left(t\right);\;\beta_{OD}\left(0\right)=\beta_{OD}^{0},$$

$${\dot{\beta }}_{HD}\left(t;m\right):=\left({\tilde{\lambda }}_{m}\left(1-\frac{\beta_{HD}^{m}\left(t\right)}{\beta_{HDmax}}\right)-\left(\frac{O_{m}^{v}}{N_{m}^{O}}+\frac{D_{m}^{T}}{N_{m}^{D}}\right)\right)\beta_{HD}^{m}\left(t\right);\;\beta_{HD}\left(0\right)=\beta_{HD}^{0}.$$

These define the dynamics of the disease transmission rates from sheep to dogs, dogs to sheep, and dogs to humans, respectively. In these three dynamic laws, it is observed that, when control measures are discontinued, the relative change in the transmission rate becomes positive, meaning that the disease transmission rate increases and returns to its initial value. Conversely, if control measures are maintained, the relative change in the transmission rate becomes negative, indicating a decrease in the disease transmission rate.

In order to qualitatively analyze the impact of activities developed in programs to control the transmission and spread of echinococcosis between peri-urban, urban, and rural areas of an environment, the system of ordinary differential equations was solved numerically using a computational code that utilized the internal function ode45 (based on a Runge–Kutta-type numerical method) of MATLAB version R2022a [26]. The numerical simulations were performed with the values recorded in Table 1 and Table 2 (see Section 2.1), which correspond to the parameters and initial data (see Table 3), respectively. To exemplify the transmission and propagation of the disease in humans and the phenomenon of adult cases inadvertently created during youth due to undetected disease, a simulation of its dynamics over a 20-year period was conducted.

## 3. Results

### 3.1. Simulations for Disease Transmission Rate

**Figure 5:**
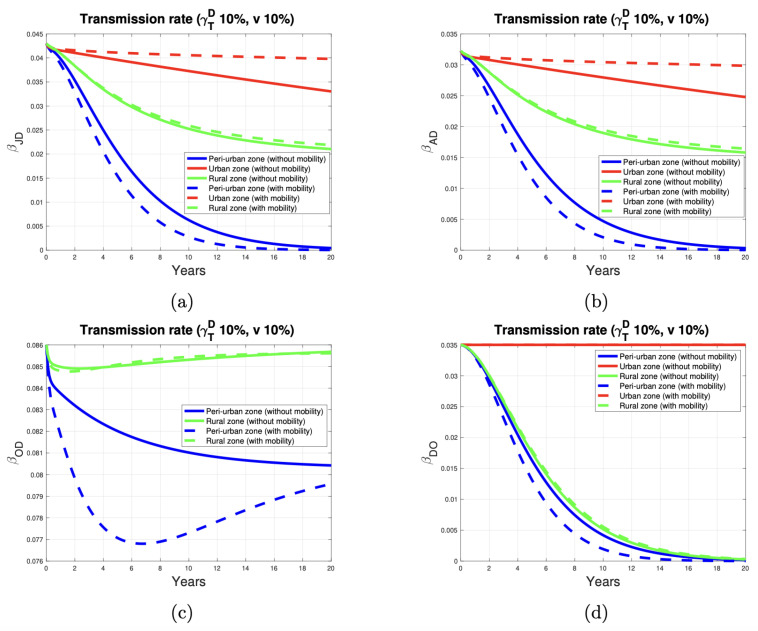
*E. granulosus* transmission rate in peri-urban (blue curve), urban (red curve), and rural (green curve) areas. (**a**–**d**) show the parasite transmission rate from dogs to juvenile humans, dogs to adult humans, dogs to sheep, and sheep to dogs, respectively.

Figure 5a presents the results of a numerical simulation. It illustrates the variability in the disease transmission rate from infected dogs to juveniles. These results follow the implementation of a control program involving the deworming and vaccination of 10% of the dog and sheep population, respectively. When host mobility parameters between the three zones are absent, the transmission rate (depicted by the continuous curve) declines more rapidly in the peri-urban zone and more gradually in the urban zone. In the rural zone, the transmission rate decreases in a pattern consistent with the trends observed in the other two zones. When we incorporate mobility into the model, the simulations clearly show that the transmission rate in the three zones is qualitatively similar to the results obtained without considering mobility. However, the transmission rate is higher in the urban zone and lower in the peri-urban zone. In the rural zone, the simulations with and without the mobility factor yield comparable results. In the urban zone, we have assumed, based on [27], that the infestation rate is zero. This is because the transmission rate’s dynamics depend solely on the proportion of deworming of dogs, and we have not considered sheep in this zone. In relative terms, the disease transmission rate stays near its threshold value in the rural zone. This is based on the assumption (from [27]) that the infestation rate is higher in rural areas compared to peri-urban zones. Consequently, the transmission rate tends toward higher restoration in the rural zone. However, implementing control measures targeting 10% of the dog and sheep population leads to a decline in the transmission rate over the 20-year simulation period in both zones.

Figure 5b illustrates the variability in the transmission rate of the disease from dogs to adult humans. In qualitative terms, the description of these curves is analogous to that of the previous scenario.

Figure 5c illustrates that, in the rural area, the rate of transmission of the disease from dogs to sheep reaches its threshold value over time. This behavior simulates a scenario in which the infestation rate is higher in this area. To reduce this rate, it is essential to increase the proportion of dewormed dogs to decontaminate the environment of dog feces contaminated with *E. granulosus*. In the peri-urban zone, the same proportion of dewormed dogs decreases the transmission rate by about 7% at the end of the simulation. The lower prevalence of the disease in dogs (as indicated by the infestation index) in the peri-urban area results in a reduced transmission rate compared to the rural area. Deworming 10% of the peri-urban dog population effectively reduces disease transmission to sheep.

As illustrated in Figure 5d, in urban areas, the transmission rate remains constant because the presence of sheep has not been considered. In contrast, in peri-urban and rural areas, the restoration of the transmission rate to its threshold value depends on the proportional mortality by EC in sheep. This proportion decreases as the proportion of vaccinated sheep increases because it is assumed that the vaccine prevents sheep from dying due to the disease.

### 3.2. The Impact of the Control Program on Humans

**Figure 6:**
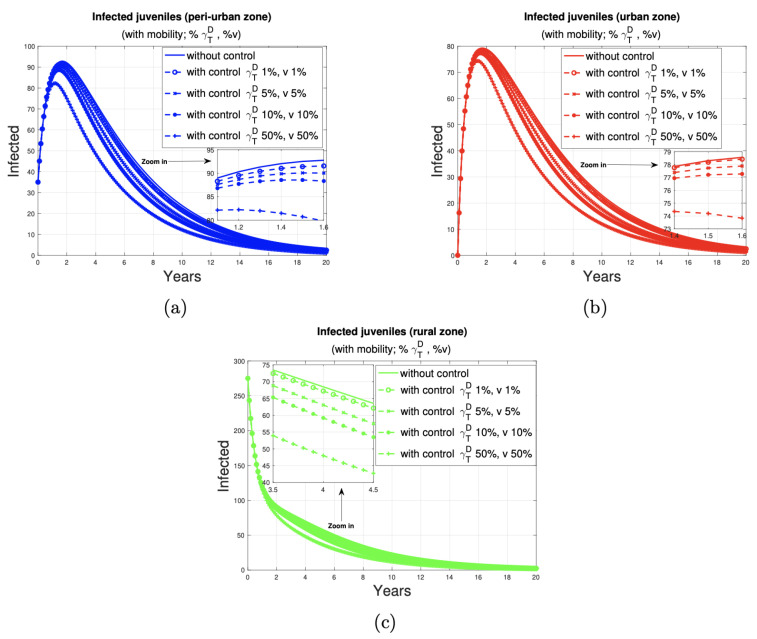
Impact of the dog and sheep disease control program on the population of humans juveniles in peri-urban (blue), urban (red), and rural (green) areas. Figure 6 (**a**), (**b**) and (**c**) graphically shows the number of juveniles infected by the disease in peri-urban, urban and rural areas, respectively, with and without a dog and ovine disease control program.

Figure 6 illustrates the 20-year progression of human juvenile infections across peri-urban, urban, and rural zones. The simulations include control programs targeting 1%, 5%, 10%, and 50% of the dog and sheep populations (marked by circles, x symbols, dots, and plus symbols, respectively).

**Figure 7:**
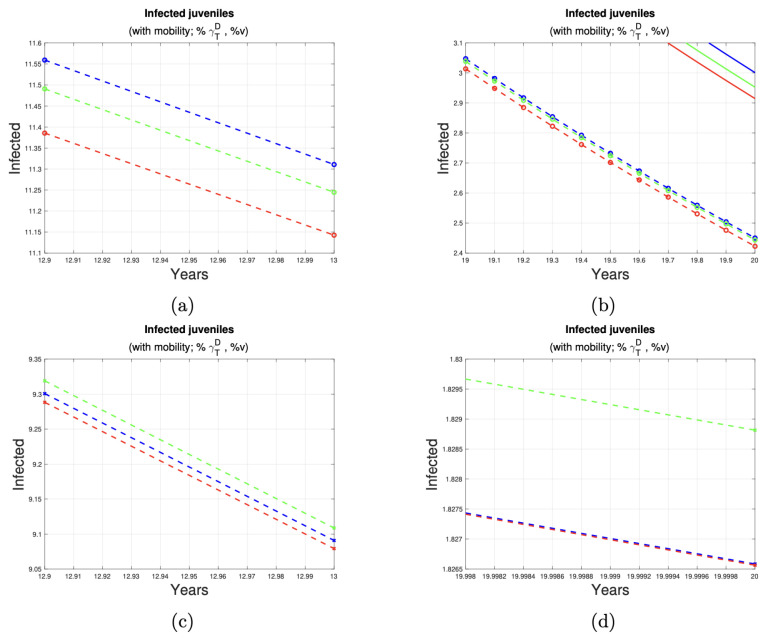
Reduction in the number of juvenile cases. (**a**,**b**) shows the reduction in cases juveniles in the last seven years of implementation of control measures at 1% (see first row of Table 4) of the dog and sheep population. (**c**,**d**) shows the reduction in cases juveniles in the last seven years of implementation of control measures at 5% (see second row of Table 4) of the dog and sheep population. The solid and dotted lines represent the number of cases of the disease in juvenile humans, without and with a program to control the disease in dogs and sheep, respectively.

**Figure 8:**
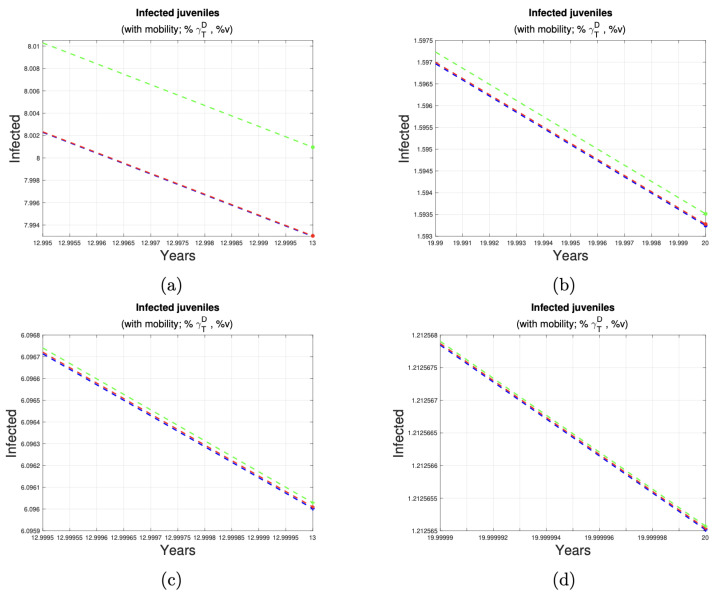
Reduction in the number of juvenile cases. (**a**,**b**) shows the reduction in cases juveniles in the last seven years of implementation of control measures to 10% (third row of Table 4) of the dog and sheep population. (**c**,**d**) shows the reduction in cases juveniles in the last seven years of implementation of control measures to 50% (fourth row of Table 4) of the dog and sheep population.

Figure 7 and Figure 8 depict the impact of varying deworming and vaccination rates on the prevalence of infection in juvenile humans. A clear trend of infection reduction is observed, particularly between years 13 and 20, as larger proportions of treated animals are present. Detailed reductions are outlined in Table 4.

To describe the impact of deworming dogs and vaccinating sheep at the different rates, we examined the behavior of juvenile human infections in the short term (2 years), medium term (8 years), and long term (20 years) (see Table 5).

- In the short term, the urban area has the lowest number of affected individuals in all disease control scenarios. An increase in the control ratio from 1% to 5% is associated with a reduction in the number of cases in the peri-urban, urban, and rural areas, with a decrease of three, one, and one cases, respectively. Similarly, an increase in the proportion of controlled animals from 1% to 10% resulted in a reduction of five, two, and three cases in these areas, respectively. Furthermore, an increase in control from 1% to 50% of the proportion of dogs and sheep results in a reduction of 16, 7, and 11 cases, respectively, in the peri-urban, urban, and rural zones.
- In the medium term, the lowest number of human juvenile infections is observed in the urban area when 1%, 5%, and 10% of the dog and sheep populations, respectively, are subjected to deworming and vaccination. When the control ratio is set at 50% of the population, the impact on each zone is consistent, with 19 cases observed in each.
- In the long term, the control of 1%, 5%, and 10% of the dog and sheep population results in a reduction to two cases in each zone. When 50% of the population is dewormed and vaccinated, the number of cases in all zones is reduced to one.

### 3.3. Effectivity Index

By definition, the effectivity of a control measure implemented in the population that transmits the infection can be measured by the index that represents the ratio between the number of infections after the control and before the control, called the control effectivity index, $\theta_{c}$.

**Figure 9:**
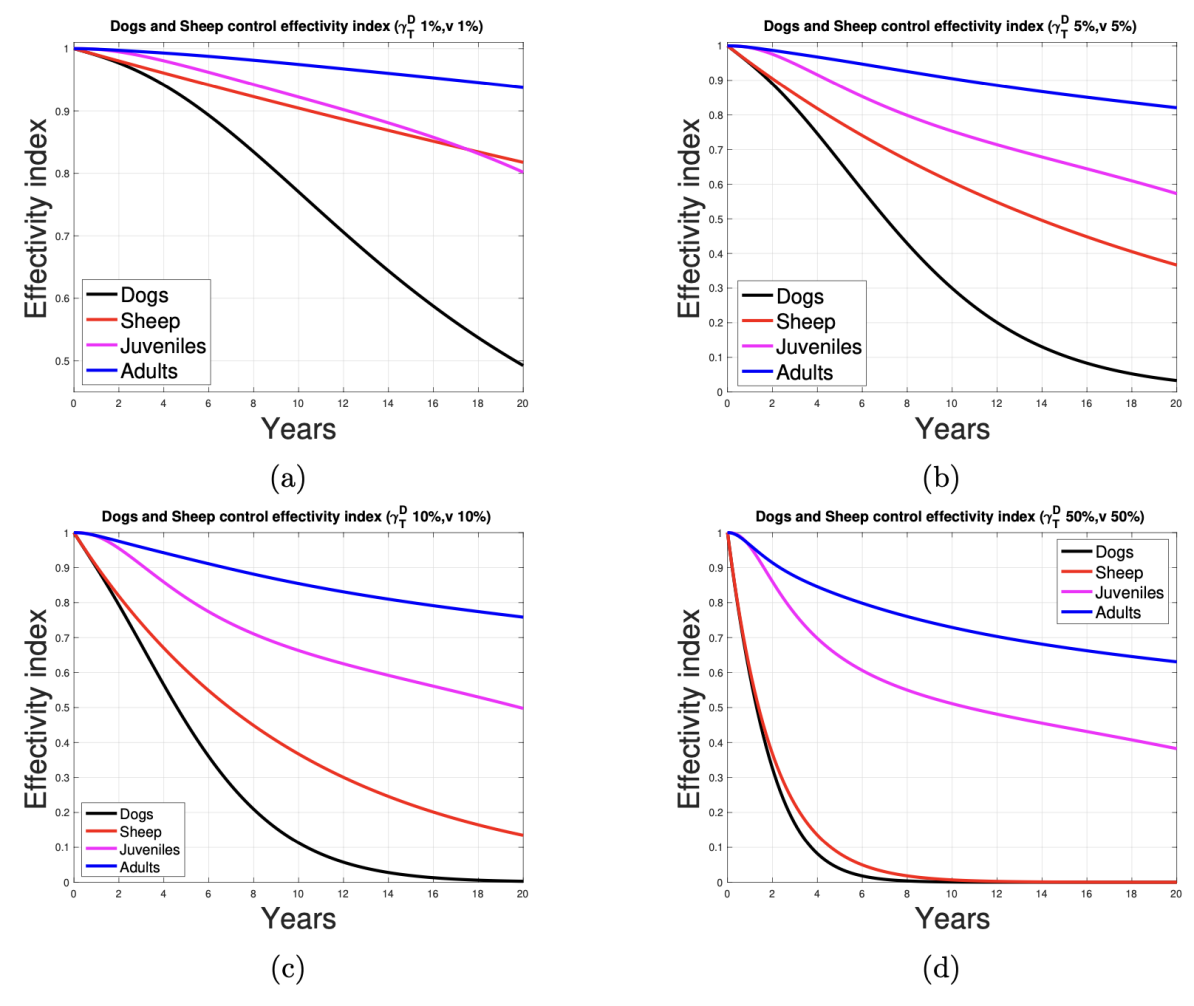
Deworming and vaccination control measures at (**a**) 1%, (**b**) 5%, (**c**) 10%, and (**d**) 50% of the ratio of animals and their effectivity in dogs, sheep, and humans.

Figure 9 illustrates the effectivity index for the activities of a control program for the populations of dogs and sheep. It can be observed that the activity directed at the dog, in which 1%, 5%, and 10% of the proportion of dogs and sheep, respectively, are dewormed and vaccinated, is the most effective. The graphics indicate that developing control activities at 5% (red and black curves are concave) of the animal population results in a faster decrease in the indicator than when it is developed in 1% (red and black curves are convex) of them. For humans, the indicator shows the most significant improvement when control activities target 50% of the dog and sheep population. Even with 10% control, a notable reduction of approximately half in human infections is achieved over 20 years (effectivity index ≈ 0.55).

### 3.4. Sensitivity Analysis of the Basic Reproductive Number and the Target Reproductive Number

**Figure 10:**
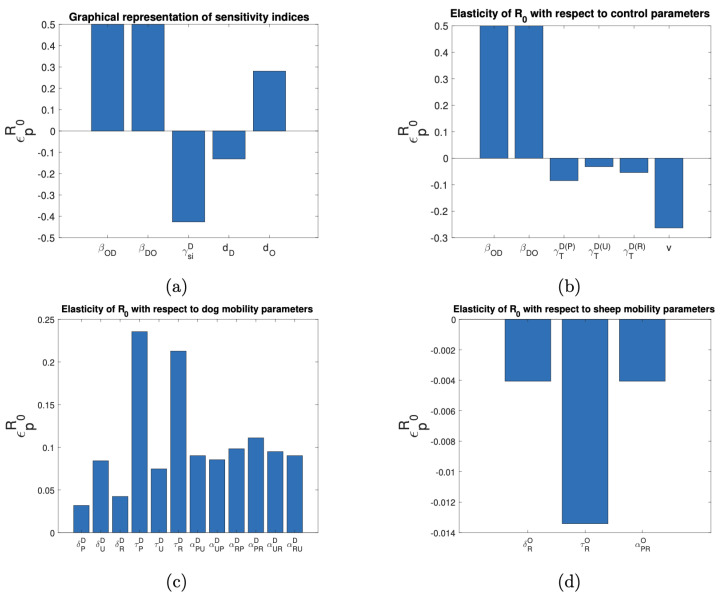
Elasticity indices on $R_{0}$ with respect to the parameters of (**a**) epidemiological, (**b**) control measures, (**c**) dog mobility, and (**d**) sheep mobility.

As $R_{0}$ is a critical parameter for the study of infectious diseases, we proceeded to calculate this intrinsic parameter of our mathematical model under the assumption of keeping the disease transmission rates constant using the next-generation matrix technique [28]. The expression found for $R_{0}$ is difficult to interpret biologically, but we were able to perform the sensitivity analysis with respect to the parameters using the elasticity indices [29]. Figure 10b illustrates that an increase in the disease transmission rate between dogs and sheep raises the reproductive number ($R_{0}$), whereas higher deworming and vaccination rates reduce $R_{0}$. For example, a 10% increase in $\beta_{OD}$ or $\beta_{DO}$ is associated with a 5% increase in $R_{0}$, and a 10% increase in $\gamma_{T}^{D(P)}$ is associated with a 0.8% decrease in $R_{0}$. Moreover, a 10% increase in *v* results in a 2.6% decrease in $R_{0}$. As illustrated in Figure 10c,d, the mobility parameters that impact $R_{0}$ most significantly are those that depend on the average residence times of dogs and sheep from rural areas in other areas.

Building on the work of Shuai et al. [30], Saldaña and Barradas [31], and González-Galeano et al. [32], and assuming constant transmission rates, we calculated the target reproductive number to evaluate vaccination and deworming strategies for disease control. This approach enabled a partial study of vaccination and deworming measures for disease control.

**Figure 11:**
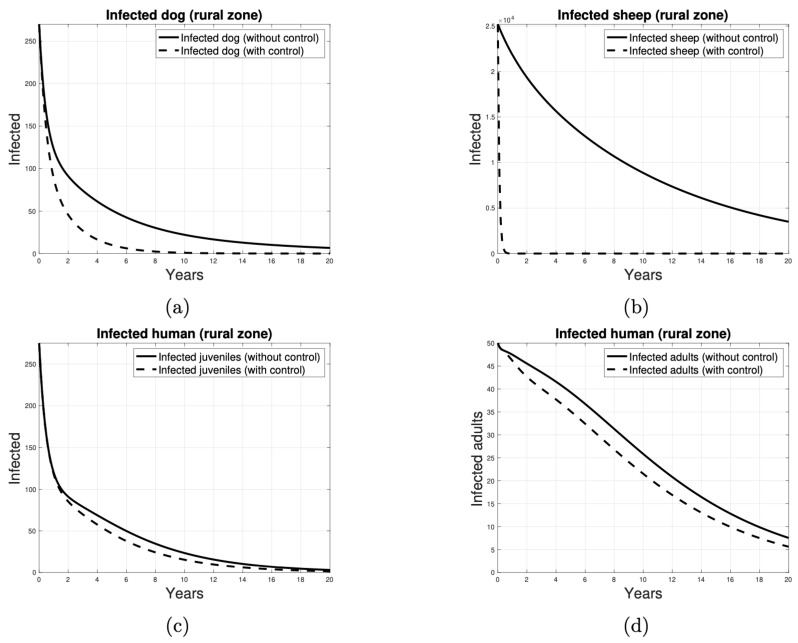
Number of individuals infected over time, when the control strategy to reduce parasite transmission is to vaccinate the sheep. The infected individuals in (**a**) are dogs, in (**b**) are sheep, in (**c**) are juvenile humans and in (**d**) are adult humans. The solid curves indicate the absence of control and the dashed curves indicate the presence of control.

In the next-generation matrix that we have obtained for our compartmental model (see Appendix A.2), each entry in the fifth column corresponds to the average number of new infections in dogs that can be produced by an infected sheep from the rural area introduced into the peri-urban and rural areas, respectively. Consequently, the target set is identified as $S_{v}=\left\{\left(1,5\right),\left(3,5\right)\right\}$. Thus, to calculate $\Gamma_{v},$ the target reproductive number of the control strategy designed to reduce the transmission of the disease from sheep to dogs by vaccination, the partition of the next-generation matrix detailed in Appendix A.3 was considered. The results of a vaccination-based control program are shown in Figure 11. As illustrated in Figure 11a,b, the disease exhibits a tendency to be eradicated in dog and sheep populations from year 8 and shortly before year 1, respectively. Figure 11c suggests that, between years 2 and 14, the number of cases in human juveniles is consistently lower when the vaccination strategy is implemented. A similar pattern is observed in Figure 11d, which indicates that, beginning in year 1, the number of cases in adult humans is reduced when sheep are vaccinated.

**Figure 12:**
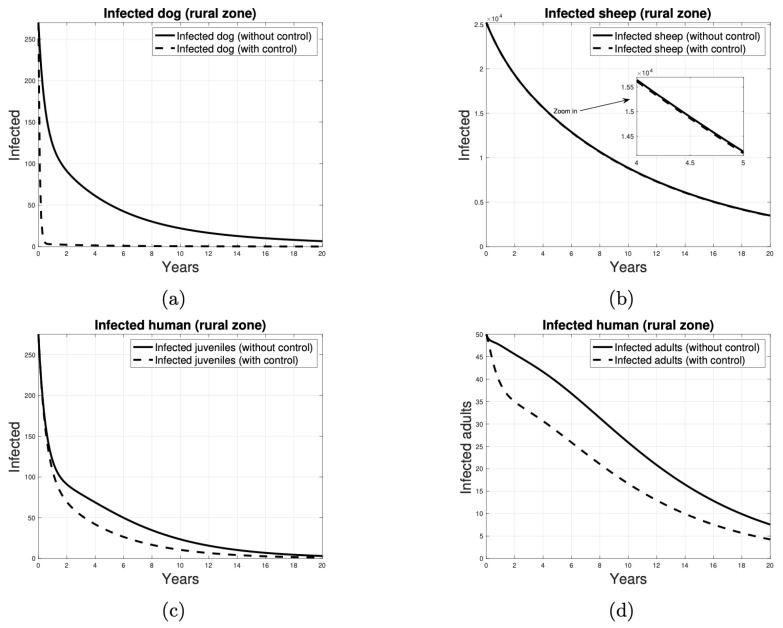
Number of individuals infected over time, when the control strategy to reduce parasite transmission is to deworming the dogs. The infected individuals in (**a**) are dogs, in (**b**) are sheep, in (**c**) are juvenile humans and in (**d**) are adult humans. Continuous curves represent scenarios without control measures, while dashed curves represent scenarios with the implementation of control measures.

Similarly, the target reproductive number of the control strategy designed to reduce the transmission of the disease from dogs to sheep through the application of deworming measures takes into account the target set $S_{\gamma_{T}^{D}}=\left\{\left(4,3\right),\left(5,3\right)\right\}$. Figure 12 presents the results of a deworming-based control program. As illustrated in Figure 12a, the disease tends to be eradicated in dogs from year 1. However, this strategy does not suggest that the disease is eliminated in sheep, potentially due to the high number of infected sheep. Figure 12c,d illustrate that human cases begin to decline earlier in the second strategy and at a greater rate.

### 3.5. The Impact of Prevention on Humans

**Figure 13:**
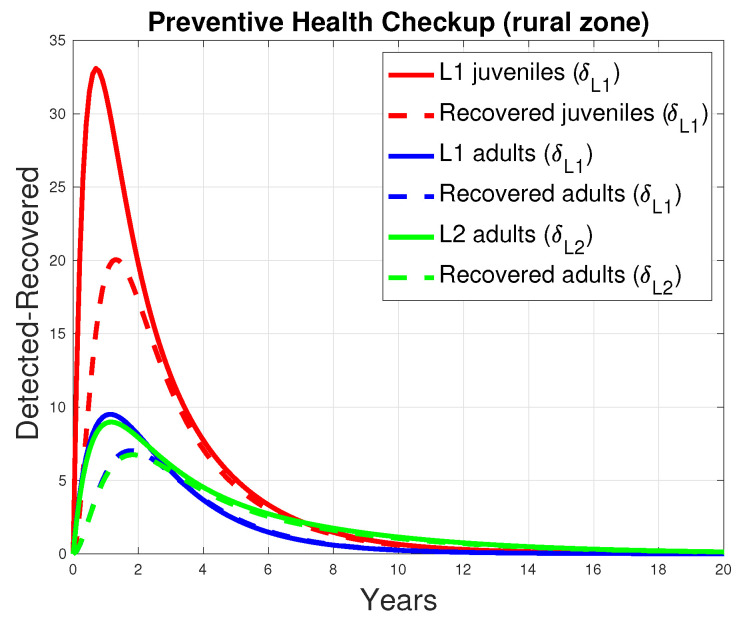
Relationship between early detection and recovery from the disease in humans. The continuous curves represent the number of patients screened for early detection, and the dashed curves represent the number of patients who recovered after receiving a treatment defined by early detection.

In the mathematical model, a preventive screening option was incorporated to enable the early detection of infections in the human population, aiming to reduce the need for surgical intervention (see Figure 3). Figure 13 presents the outcomes of subjecting juveniles and adults to early detection screening at two levels ($\delta_{L1}$ and $\delta_{L2}$), where positive results lead to pharmacological treatment (level 1) or surgical intervention (level 2), as appropriate. Level 2 screening was conducted on 10% of the adult population in an at-risk area (χ=0.1). The simulation results indicate a strong correlation between early disease detection and patient recovery outcomes.

## 4. Discussion

Given that the therapeutic options for CE in humans are not sufficiently compelling [33] and that the monetary burden associated with the disease in animals and humans is significant, strategies to prevent and control CE are more rational [34]. This underscores the need for tools to enhance epidemiological surveillance, as well as prevention and control strategies. To implement effective prevention and control programs for this disease, establishing a baseline to determine the prevalence of echinococcosis in dogs, sheep, and humans is essential [9]. Previous studies by Lutz et al. [35], Acosta-Jamett et al. [19], Lorca et al. [36], and Alvarez et al. [27] have provided recommendations for the control of echinococcosis based on data and results from field and laboratory studies conducted at a given time, with a descriptive approach. However, the mechanisms of transmission of this disease in hosts were not explicitly considered. This approach does not allow for the monitoring and evaluation of disease mitigation or control measures that have not been implemented. To complement this approach in the task of analyzing prevention and control programs for this disease, mathematical modeling is a tool that allows the tracking and evaluation of the impact of a measure that has not yet been implemented in the study population. In the study by Wu et al. [13], through a sensitivity analysis of the proposed model, it was found that the human intervention coefficient (deworming and disposal of buried dog feces) was the most influential control parameter affecting the basic reproductive number. Similarly, Birhan et al. [14] evaluated two disease control strategies: sheep vaccination and environmental disinfection or cleaning. They concluded that sheep vaccination was the most effective strategy in eradicating the disease. However, Birhan et al. [14] recommend the inclusion of a dog-centered control strategy to eliminate echinococcosis transmission. The significant decrease in $R_{0}$ observed in Figure 10b highlights the fundamental role of sheep vaccination as the most effective strategy in reducing the basic reproductive number ($R_{0}$) of echinococcosis. Therefore, these results are consistent with the results of this study. Moreover, our analysis highlights that host mobility significantly influences the dynamics of the disease transmission rates, contributing to the spread of CE across peri-urban, urban, and rural areas. On the one hand, our results based on the simulation of the control of CE in adult humans and sheep through deworming dogs (see Figure 12b,d) could describe the persistence of the disease in South American countries, where the population maintains the custom of feeding dogs with offal, as indicated by Poggio et al. in their study [37]. On the other hand, when our model control is carried out by vaccinating sheep (see Figure 11a), in the medium term, there are still infected dogs, as indicated by [37]. This same simulation of the control strategy (see Figure 11b) based on sheep vaccination shows, qualitatively, in the short term, a small delay before the effect of the decrease in the number of infected sheep occurs, as pointed out by Liu et al. in [38], who justify this in terms of the time that it takes for the number of mature cysts to decrease after vaccination. In the present study, a strategic approach has been implemented in the mathematical model, whereby simulations have been obtained from data from endemic areas, and not from one in particular. This choice could, in light of the findings of Borhani et al. [39], constitute a limitation when implementing an effective control program in a specific endemic area. However, this methodological approach was useful in understanding the role of various factors, such as host mobility, sheep vaccination, dog deworming, and the early detection of the disease in humans, in the phenomenon of *E. granulosus’* spread and control.

## 5. Conclusions

In this study, a mathematical model was developed and analyzed to describe the dynamics of transmission and spread of the parasite *Echinococcus granulosus* among humans, sheep, and dogs in peri-urban, urban, and rural areas. The model incorporates the transmission dynamics of the disease across its hosts. Using the effectiveness index, it was demonstrated that simultaneous dog deworming and sheep vaccination programs are effective in reducing the populations of infected hosts. Assuming constant disease transmission rates, a next-generation matrix was employed to calculate the basic reproductive number $R_{0}$. A sensitivity analysis, based on the elasticity indices of $R_{0}$ with respect to its parameters, revealed that the dog-to-sheep transmission rate, the residence times of dogs and sheep in other areas, and vaccination are the most influential factors affecting $R_{0}$. Furthermore, by using the target reproductive number, it was determined that the dog deworming strategy can consistently reduce human infections, while the sheep vaccination strategy provides a more favorable scenario for disease eradication in the two mammalian hosts. In addition, when including parameters in the mathematical model to characterize a preventive examination targeting at-risk populations, the simulations showed a strong correlation between early disease detection and improved patient recovery outcomes.

## Figures and Tables

**Table 1:** Definitions and values of control parameters (unit: yr^−1^).

Parameters	Description	Value	Range	Reference
$\gamma_{T}^{D}$	Rate of deworming of dogs	0.0100	0.0100–1.0000	[11]
$1/\tau_{T}$	Periodicity between deworming	0.1233	-	[4]
*v*	Rate of vaccination of sheep	0.0100	0.0100–1.0000	[11]
$\gamma_{sv}^{O}$	Rate of loss of vaccine-induced immunity of sheep	0.0365	-	[14]
$\delta_{L1}$	Detection rate L1	0.0010	0.0000–1.0000	[19]
$\delta_{L2}$	Detection rate L2	0.0010	0.0000–1.0000	[19]
$1/\tau_{L1}$	Average treatment time L1	0.2500	0.0000–1.0000	[20,21]
$1/\tau_{L1}$	Average treatment time L2	0.5000	0.0000–1.0000	[20,21]
$1/\tau_{sr}$	Average time loss of immunity	0.5000	-	Assumed

**Table 2:** Definitions and values of mobility and epidemiological parameters (unit: yr^−1^).

Parameters	Description	Value	Range	Reference
$\mu_{D}$	Dog birth rate	0.0800	0.0800–0.3212	[13,14,22,23,24]
$\mu_{O}$	Sheep birth rate	0.0833	0.0052–0.3333	[13,14,22,23,24]
$\mu_{H}$	Human birth rate	0.0141	0.0141–0.4161	[13,14,22,23,24]
$d_{D}$	Dog death rate	0.0800	0.0800–0.3212	[13,14,22,23,24]
$d_{O}$	Sheep death rate	0.0833	0.0052–0.3333	[13,14,22,23,24]
$d_{H}$	Human death rate	0.0141	0.0141–0.4161	[13,14,22,23,24]
$d_{i}^{H}$	Human disease-related death rate	0.1460	0.0009–0.1460	[13,24]
$\beta_{DO}$	Transmission rate from sheep to dogs	0.0350	5.8 × 10^−8^–0.0350	[13,14,22,24]
$\beta_{OD}$	Transmission rate from dogs to sheep	0.0860	7.4 × 10^−8^–0.3650	[13,14,22,24]
$\beta_{JD}$	Transmission rate from dogs to human children	0.0430	4.2 × 10^−11^–0.0430	[13,14,22,24]
$\beta_{AD}$	Transmission rate from dogs to human adults	0.0323	4.2 × 10^−11^–0.0430	[13,14,22,24]
$\gamma_{ie}^{O}$	Rate at which exposed sheep progress to infected	0.1090	0.0365–0.1090	[14,22,23]
$\gamma_{ie}^{H}$	Rate at which exposed humans progress to infected	0.0714	0.0693–0.0714	[14,22,23]
$\gamma_{si}^{D}$	Rate at which infected dogs progress to susceptible	0.2500	0.2500–2.4000	[2]
$\gamma_{si}^{H}$	Rate at which infected humans receive treatment	0.5000	0.0500–0.5000	[25]
$1/\tau_{Z}^{X}$	Average time that a host *X* from zone *Z* stays in another zone	0.5000	-	Assumed
$\delta_{Z}^{X}$	Rate of exit of host *X* from area *Z*	0.3030	-	Assumed
$\alpha_{AB}^{X}$	Proportion of hosts *X* moving from area *B* to area *A*	0.5000	0.0000–1.0000	Assumed

**Table 3:** Initial host values according to epidemiological stages and area.

	*s* Susceptible	*i* Infected	*T* Treated	*v* Vaccinated	*u* Undetected	L1 Treated Level 1	L2 Treated Level 2	*r* Recovered
Peri-urban								
$D_{P}\left(0\right)$	500	20	100	-	-	-	-	-
$O_{P}\left(0\right)$	8680	594	-	100	-	-	-	-
$J_{P}\left(0\right)$	647	-	-	-	35	0	0	0
$A_{P}\left(0\right)$	658	-	-	-	35	0	0	0
Urban								
$D_{U}\left(0\right)$	4000	50	1000	-	-	-	-	-
$J_{U}\left(0\right)$	25,000	-	-	-	0	0	0	0
$A_{U}\left(0\right)$	25,018	-	-	-	15	0	0	0
Rural								
$D_{R}\left(0\right)$	1500	270	150	-	-	-	-	-
$O_{R}\left(0\right)$	70,000	25,200	-	1000	-	-	-	-
$J_{R}\left(0\right)$	1520	-	-	-	275	0	0	0
$A_{R}\left(0\right)$	1556	-	-	-	50	0	0	0

**Table 4:** Reduction in cases in juveniles in the last seven years of implementation of control measures in dogs and sheep.

Ratio of Animals Under Control	Initial Cases (Year 13)	Final Cases (Year 20)	Case Reduction (Approximate)	Figure
1%	11	2	9	Figure 7a,b
5%	9	2	7	Figure 7c,d
10%	8	2	7	Figure 8a,b
50%	6	1	5	Figure 8c,d

**Table 5:** Timing of a control program for dogs and sheep.

Ratio of Animals Under Control	Cases (Year 2)	Cases (Year 8)	Cases (Year 20)	Zone
1%	91	33	2	Peri-urban
1%	78	28	2	Urban
1%	90	32	2	Rural
5%	88	28	2	Peri-urban
5%	77	28	2	Urban
5%	89	28	2	Rural
10%	86	25	2	Peri-urban
10%	76	25	2	Urban
10%	87	25	2	Rural
50%	75	19	1	Peri-urban
50%	71	19	1	Urban
50%	79	19	1	Rural

## Data Availability

Data are contained within the article.

## Abbreviations

The following abbreviations are used in this manuscript:

CE	Cystic Echinococcosis
WHO	World Health Organization
PAHO	Pan American Health Organization

## Appendix A. System of Ordinary Differential Equations, the Next-Generation Matrix, and the Target Reproductive Number

### Appendix A.1. Proposed Mathematical Model

$$\begin{array}{cc} {\dot{D}}_{P}^{s} & =\left(\alpha_{PR}^{D}\delta_{R}^{D}+\tau_{P}^{D}\right)D_{R}^{s}+\left(\alpha_{PU}^{D}\delta_{U}^{D}+\tau_{P}^{D}\right)D_{U}^{s}-\left(\tau_{U}^{D}+\tau_{R}^{D}+\delta_{P}^{D}\right)D_{P}^{s}\\ \; & +\mu_{D}N_{P}^{D}+\gamma_{si}^{D}D_{P}^{i}+\tau_{T}D_{P}^{T}-\left(\frac{\beta_{DO}d_{O}O_{P}^{i}}{N_{P}^{D}}+\gamma_{T}^{D}+d_{D}\right)D_{P}^{s}\\ {\dot{D}}_{P}^{i} & =\left(\alpha_{PR}^{D}\delta_{R}^{D}+\tau_{P}^{D}\right)D_{R}^{i}+\left(\alpha_{PU}^{D}\delta_{U}^{D}+\tau_{P}^{D}\right)D_{U}^{i}-\left(\tau_{R}^{D}+\tau_{U}^{D}+\delta_{P}^{D}\right)D_{P}^{i}\\ \; & +\frac{\beta_{DO}d_{O}O_{P}^{i}}{N_{P}^{D}}D_{P}^{s}-\left(d_{D}+\gamma_{si}^{D}+\gamma_{T}^{D}\right)D_{P}^{i}\\ {\dot{D}}_{P}^{T} & =\left(\alpha_{PR}^{D}\delta_{R}^{D}+\tau_{P}^{D}\right)D_{R}^{T}+\left(\alpha_{PU}^{D}\delta_{U}^{D}+\tau_{P}^{D}\right)D_{U}^{T}-\left(\tau_{U}^{D}+\tau_{R}^{D}+\delta_{P}^{D}\right)D_{P}^{T}\\ \; & +\gamma_{T}^{D}D_{P}^{s}+\gamma_{T}^{D}D_{P}^{i}-\left(d_{D}+\tau_{T}\right)D_{P}^{T}\\ {\dot{D}}_{U}^{s} & =\left(\alpha_{UP}^{D}\delta_{P}^{D}+\tau_{U}^{D}\right)D_{P}^{s}+\left(\alpha_{UR}^{D}\delta_{R}^{D}+\tau_{U}^{D}\right)D_{R}^{s}-\left(\tau_{R}^{D}+\tau_{P}^{D}+\delta_{U}^{D}\right)D_{U}^{s}\\ \; & +\mu_{D}N_{U}^{D}+\gamma_{si}^{D}D_{U}^{i}+\tau_{T}D_{U}^{T}-\left(d_{D}+\gamma_{T}^{D}\right)D_{U}^{s}\\ {\dot{D}}_{U}^{i} & =\left(\alpha_{UP}^{D}\delta_{P}^{D}+\tau_{U}^{D}\right)D_{P}^{i}+\left(\alpha_{UR}^{D}\delta_{R}^{D}+\tau_{U}^{D}\right)D_{R}^{i}-\left(\tau_{P}^{D}+\tau_{R}^{D}+\delta_{U}^{D}\right)D_{U}^{i}\\ \; & -\left(d_{D}+\gamma_{si}^{D}+\gamma_{T}^{D}\right)D_{U}^{i}\\ {\dot{D}}_{U}^{T} & =\left(\alpha_{UP}^{D}\delta_{P}^{D}+\tau_{U}^{D}\right)D_{P}^{T}+\left(\alpha_{UR}^{D}\delta_{R}^{D}+\tau_{U}^{D}\right)D_{R}^{T}-\left(\tau_{P}^{D}+\tau_{R}^{D}+\delta_{U}^{D}\right)D_{U}^{T}\\ \; & +\gamma_{T}^{D}D_{U}^{s}+\gamma_{T}^{D}D_{U}^{i}-\left(d_{D}+\tau_{T}\right)D_{U}^{T}\\ {\dot{D}}_{R}^{s} & =\left(\alpha_{RP}^{D}\delta_{P}^{D}+\tau_{R}^{D}\right)D_{P}^{s}+\left(\alpha_{RU}^{D}\delta_{U}^{D}+\tau_{R}^{D}\right)D_{U}^{s}-\left(\tau_{P}^{D}+\tau_{U}^{D}+\delta_{R}^{D}\right)D_{R}^{s}\\ \; & +\mu_{D}N_{R}^{D}+\gamma_{si}^{D}D_{R}^{i}+\tau_{T}D_{R}^{T}-\left(\frac{\beta_{DO}d_{O}O_{R}^{i}}{N_{R}^{D}}+d_{D}+\gamma_{T}^{D}\right)D_{R}^{s}\\ {\dot{D}}_{R}^{i} & =\left(\alpha_{RP}^{D}\delta_{P}^{D}+\tau_{R}^{D}\right)D_{P}^{i}+\left(\alpha_{RU}^{D}\delta_{U}^{D}+\tau_{R}^{D}\right)D_{U}^{i}-\left(\tau_{P}^{D}+\tau_{U}^{D}+\delta_{R}^{D}\right)D_{R}^{i}\\ \; & +\frac{\beta_{DO}d_{O}O_{R}^{i}}{N_{R}^{D}}D_{R}^{s}-\left(d_{D}+\gamma_{si}^{D}+\gamma_{T}^{D}\right)D_{R}^{i}\\ {\dot{D}}_{R}^{T} & =\left(\alpha_{RP}^{D}\delta_{P}^{D}+\tau_{R}^{D}\right)D_{P}^{T}+\left(\alpha_{RU}^{D}\delta_{U}^{D}+\tau_{R}^{D}\right)D_{U}^{T}-\left(\tau_{P}^{D}+\tau_{U}^{D}+\delta_{R}^{D}\right)D_{R}^{T}\\ \; & +\gamma_{T}^{D}D_{R}^{s}+\gamma_{T}^{D}D_{R}^{i}-\left(d_{D}+\tau_{T}\right)D_{R}^{T} \end{array}$$

$$\begin{array}{cc} {\dot{O}}_{P}^{s} & =\alpha_{PR}^{O}\delta_{R}^{O}O_{R}^{s}-\tau_{R}^{O}O_{P}^{s}+\mu_{O}N_{P}^{O}+\gamma_{sv}^{O}-\left(v+\frac{\beta_{OD}D_{P}^{i}}{N_{P}^{O}}+d_{O}\right)O_{P}^{s}\\ {\dot{O}}_{P}^{i} & =\alpha_{PR}^{O}\delta_{R}^{O}O_{R}^{i}-\tau_{R}^{O}O_{P}^{i}+\frac{\beta_{OD}D_{P}^{i}O_{P}^{s}}{N_{P}^{O}}-\left(v+d_{O}\right)O_{P}^{i}\\ {\dot{O}}_{P}^{v} & =\alpha_{PR}^{O}\delta_{R}^{O}O_{R}^{v}-\tau_{R}^{O}O_{P}^{v}+\left(O_{P}^{s}+O_{P}^{i}\right)v-\left(\gamma_{sv}^{O}+d_{O}\right)O_{P}^{v}\\ {\dot{O}}_{R}^{s} & =\tau_{R}^{O}O_{P}^{s}-\alpha_{PR}^{O}\delta_{R}^{O}O_{R}^{s}+\mu_{O}N_{R}^{O}+\gamma_{sv}^{O}O_{R}^{v}-\left(\frac{\beta_{OD}D_{R}^{i}}{N_{R}^{O}}+d_{O}+v\right)O_{R}^{s}\\ {\dot{O}}_{R}^{i} & =\tau_{R}^{O}O_{P}^{i}-\alpha_{PR}^{O}\delta_{R}^{O}O_{R}^{i}+\frac{\beta_{OD}D_{R}^{i}O_{R}^{s}}{N_{R}^{O}}-\left(v+d_{O}\right)O_{R}^{i}\\ {\dot{O}}_{R}^{v} & =\tau_{R}^{O}O_{P}^{v}-\alpha_{PR}^{O}\delta_{R}^{O}O_{R}^{v}+\left(O_{R}^{s}+O_{R}^{i}\right)v-\left(\gamma_{sv}^{O}+d_{O}\right)O_{R}^{v} \end{array}$$

$$\begin{array}{cc} {\dot{J}}_{P}^{s} & =\left(\alpha_{PR}^{J}\delta_{R}^{J}+\tau_{P}^{J}\right)J_{R}^{s}+\left(\alpha_{PU}^{J}\delta_{U}^{J}+\tau_{P}^{J}\right)J_{U}^{s}-\left(\tau_{R}^{J}+\tau_{U}^{J}+\delta_{P}^{J}\right)J_{P}^{s}\\ \; & +\mu_{H}N_{P}^{H}+\tau_{sr}J_{P}^{r}-\left(\frac{\beta_{JD}D_{P}^{i}}{N_{P}^{H}}+\tau +d_{H}\right)J_{P}^{s}\\ {\dot{J}}_{P}^{u} & =\left(\alpha_{PR}^{J}\delta_{R}^{J}+\tau_{P}^{J}\right)J_{R}^{u}+\left(\alpha_{PU}^{J}\delta_{U}^{J}+\tau_{P}^{J}\right)J_{U}^{u}-\left(\tau_{U}^{J}+\tau_{R}^{J}+\delta_{P}^{J}\right)J_{P}^{u}\\ \; & +\frac{\beta_{JD}D_{P}^{i}}{N_{P}^{H}}J_{P}^{s}-\left(\tau +d_{H}+\delta_{L1}\right)J_{P}^{u}\\ {\dot{J}}_{P}^{L1} & =\delta_{L1}J_{P}^{u}-\left(\tau_{L1}+d_{H}\right)J_{P}^{L1}\\ {\dot{J}}_{P}^{r} & =\tau_{L1}J_{P}^{L1}-\left(\tau_{sr}+\tau +d_{H}\right)J_{P}^{r}\\ {\dot{J}}_{U}^{s} & =\left(\alpha_{UP}^{J}\delta_{P}^{J}+\tau_{U}^{J}\right)J_{P}^{s}+\left(\alpha_{UR}^{J}\delta_{R}^{J}+\tau_{U}^{J}\right)J_{R}^{s}-\left(\tau_{R}^{J}+\tau_{P}^{J}+\delta_{U}^{J}\right)J_{U}^{s}\\ \; & +\mu_{H}N_{U}^{H}+\tau_{sr}J_{U}^{r}-\left(\frac{\beta_{JD}D_{U}^{i}}{N_{U}^{H}}+\tau +d_{H}\right)J_{U}^{s}\\ {\dot{J}}_{U}^{u} & =\left(\alpha_{UP}^{J}\delta_{P}^{J}+\tau_{U}^{J}\right)J_{P}^{u}+\left(\alpha_{UR}^{J}\delta_{R}^{J}+\tau_{U}^{J}\right)J_{R}^{u}-\left(\tau_{R}^{J}+\tau_{P}^{J}+\delta_{U}^{J}\right)J_{U}^{u}\\ \; & +\frac{\beta_{JD}D_{U}^{i}}{N_{U}^{H}}J_{U}^{s}-\left(\tau +d_{H}+\delta_{L1}\right)J_{U}^{u}\\ {\dot{J}}_{U}^{L1} & =\delta_{L1}J_{U}^{u}-\left(\tau_{L1}+d_{H}\right)J_{U}^{L1}\\ {\dot{J}}_{U}^{r} & =\tau_{L1}J_{U}^{L1}-\left(\tau_{sr}+\tau +d_{H}\right)J_{U}^{r}\\ {\dot{J}}_{R}^{s} & =\left(\alpha_{RP}^{J}\delta_{P}^{J}+\tau_{R}^{J}\right)J_{P}^{s}+\left(\alpha_{RU}^{J}\delta_{U}^{J}+\tau_{R}^{J}\right)J_{U}^{s}-\left(\tau_{P}^{J}+\tau_{U}^{J}+\delta_{R}^{J}\right)J_{R}^{s}\\ \; & +\mu_{H}N_{R}^{H}+\tau_{sr}J_{R}^{r}-\left(\frac{\beta_{JD}D_{R}^{i}}{N_{R}^{H}}+\tau +d_{H}\right)J_{R}^{s}\\ {\dot{J}}_{R}^{u} & =\left(\alpha_{RP}^{J}\delta_{P}^{J}+\tau_{R}^{J}\right)J_{P}^{u}+\left(\alpha_{RU}^{J}\delta_{U}^{J}+\tau_{R}^{J}\right)J_{U}^{u}-\left(\tau_{P}^{J}+\tau_{U}^{J}+\delta_{R}^{J}\right)J_{R}^{u}\\ \; & +\frac{\beta_{JD}D_{R}^{i}}{N_{R}^{H}}J_{R}^{s}-\left(\tau +d_{H}+\delta_{L1}\right)J_{R}^{u}\\ {\dot{J}}_{R}^{L1} & =\delta_{L1}J_{R}^{u}-\left(\tau_{L1}+d_{H}\right)J_{R}^{L1}\\ {\dot{J}}_{R}^{r} & =\tau_{L1}J_{R}^{L1}-\left(\tau_{sr}+\tau +d_{H}\right)J_{R}^{r} \end{array}$$

$$\begin{array}{cc} {\dot{A}}_{P}^{s} & =\left(\alpha_{PR}^{A}\delta_{R}^{A}+\tau_{P}^{A}\right)A_{R}^{s}+\left(\alpha_{PU}^{A}\delta_{U}^{A}+\tau_{P}^{A}\right)A_{U}^{s}-\left(\tau_{R}^{A}+\tau_{U}^{A}+\delta_{P}^{A}\right)A_{P}^{s}\\ \; & +\tau J_{P}^{s}+\tau_{sr}A_{P}^{r}-\left(\frac{\beta_{AD}D_{P}^{i}}{N_{P}^{H}}+d_{H}\right)A_{P}^{s}\\ {\dot{A}}_{P}^{u} & =\left(\alpha_{PR}^{A}\delta_{R}^{A}+\tau_{P}^{A}\right)A_{R}^{u}+\left(\alpha_{PU}^{A}\delta_{U}^{A}+\tau_{P}^{A}\right)A_{U}^{u}-\left(\tau_{R}^{A}+\tau_{U}^{A}+\delta_{P}^{A}\right)A_{P}^{u}\\ \; & +\frac{\beta_{AD}D_{P}^{i}}{N_{P}^{H}}A_{P}^{s}+\tau J_{P}^{u}-\left(d_{H}+\chi \delta_{L2}+\left(1-\chi \right){\tilde{\delta }}_{L2}+\delta_{L1}\right)A_{P}^{u}\\ {\dot{A}}_{P}^{L1} & =\delta_{L1}A_{P}^{u}-\left(\tau_{L1}+d_{H}\right)A_{P}^{L1}\\ {\dot{A}}_{P}^{L2} & =\left(\chi \delta_{L2}+\left(1-\chi \right){\tilde{\delta }}_{L2}\right)A_{P}^{u}-\left(\chi \tau_{L2}+\left(1-\chi \right){\tilde{\tau }}_{L2}+d_{H}\right)A_{P}^{L2}\\ {\dot{A}}_{P}^{r} & =\tau_{L1}A_{P}^{L1}+\left(\chi \tau_{L2}+\left(1-\chi \right){\tilde{\tau }}_{L2}\right)A_{P}^{L2}+\tau J_{P}^{r}-\left(\tau_{sr}+d_{H}\right)A_{P}^{r}\\ {\dot{A}}_{U}^{s} & =\left(\alpha_{UP}^{A}\delta_{P}^{A}+\tau_{U}^{A}\right)A_{P}^{s}+\left(\alpha_{UR}^{A}\delta_{R}^{A}+\tau_{U}^{A}\right)A_{R}^{s}-\left(\tau_{R}^{A}+\tau_{P}^{A}+\delta_{U}^{A}\right)A_{U}^{s}\\ \; & +\tau J_{U}^{s}+\tau_{sr}A_{U}^{r}-\left(\frac{\beta_{AD}D_{U}^{i}}{N_{U}^{H}}+d_{H}\right)A_{U}^{s}\\ {\dot{A}}_{U}^{u} & =\left(\alpha_{UP}^{A}\delta_{P}^{A}+\tau_{U}^{A}\right)A_{P}^{u}+\left(\alpha_{UR}^{A}\delta_{R}^{A}+\tau_{U}^{A}\right)A_{R}^{u}-\left(\tau_{R}^{A}+\tau_{P}^{A}+\delta_{U}^{A}\right)A_{U}^{u}\\ \; & +\frac{\beta_{AD}D_{U}^{i}}{N_{U}^{H}}A_{U}^{s}+\tau J_{U}^{u}-\left(d_{H}+\chi \delta_{L2}+\left(1-\chi \right){\tilde{\delta }}_{L2}+\delta_{L1}\right)A_{U}^{u}\\ {\dot{A}}_{U}^{L1} & =\delta_{L1}A_{U}^{u}-\left(\tau_{L1}+d_{H}\right)A_{U}^{L1}\\ {\dot{A}}_{U}^{L2} & =\left(\chi \delta_{L2}+\left(1-\chi \right){\tilde{\delta }}_{L2}\right)A_{U}^{u}-\left(\chi \tau_{L2}+\left(1-\chi \right){\tilde{\tau }}_{L2}+d_{H}\right)A_{U}^{L2}\\ {\dot{A}}_{U}^{r} & =\tau_{L1}A_{U}^{L1}+\left(\chi \tau_{L2}+\left(1-\chi \right){\tilde{\tau }}_{L2}\right)A_{U}^{L2}+\tau J_{U}^{r}-\left(\tau_{sr}+d_{H}\right)A_{U}^{r}\\ {\dot{A}}_{R}^{s} & =\left(\alpha_{RP}^{A}\delta_{P}^{A}+\tau_{R}^{A}\right)A_{P}^{s}+\left(\alpha_{RU}^{A}\delta_{U}^{A}+\tau_{R}^{A}\right)A_{U}^{s}-\left(\tau_{P}^{A}+\tau_{U}^{A}+\delta_{R}^{A}\right)A_{R}^{s}\\ \; & +\tau J_{R}^{s}+\tau_{sr}A_{R}^{r}-\left(\frac{\beta_{AD}D_{R}^{i}}{N_{R}^{H}}+d_{H}\right)A_{R}^{s}\\ {\dot{A}}_{R}^{u} & =\left(\alpha_{RP}^{A}\delta_{P}^{A}+\tau_{R}^{A}\right)A_{P}^{u}+\left(\alpha_{RU}^{A}\delta_{U}^{A}+\tau_{R}^{A}\right)A_{U}^{u}-\left(\tau_{P}^{A}+\tau_{U}^{A}+\delta_{R}^{A}\right)A_{R}^{u}\\ \; & +\frac{\beta_{AD}D_{R}^{i}}{N_{R}^{H}}A_{R}^{s}+\tau J_{R}^{u}-\left(d_{H}+\delta_{L1}+\chi \delta_{L2}+\left(1-\chi \right){\tilde{\delta }}_{L2}\right)A_{R}^{u}\\ {\dot{A}}_{R}^{L1} & =\delta_{L1}A_{R}^{u}-\left(\tau_{L1}+d_{H}\right)A_{R}^{L1}\\ {\dot{A}}_{R}^{L2} & =\left(\chi \delta_{L2}+\left(1-\chi \right){\tilde{\delta }}_{L2}\right)A_{R}^{u}-\left(\chi \tau_{L2}+\left(1-\chi \right){\tilde{\tau }}_{L2}+d_{H}\right)A_{R}^{L2}\\ {\dot{A}}_{R}^{r} & =\tau_{L1}A_{R}^{L1}+\left(\chi \tau_{L2}+\left(1-\chi \right){\tilde{\tau }}_{L2}\right)A_{R}^{L2}+\tau J_{R}^{r}-\left(\tau_{sr}+d_{H}\right)A_{R}^{r} \end{array}$$

### Appendix A.2. The Next-Generation Matrix

The following is the structure of the next-generation matrix:

$$K\left(E_{0}\right)=\left[\begin{array}{ccccccccccc} 0 & 0 & 0 & k_{1,4} & k_{1,5} & 0 & 0 & 0 & 0 & 0 & 0\\ 0 & 0 & 0 & 0 & 0 & 0 & 0 & 0 & 0 & 0 & 0\\ 0 & 0 & 0 & k_{3,4} & k_{3,5} & 0 & 0 & 0 & 0 & 0 & 0\\ k_{4,1} & k_{4,2} & k_{4,3} & 0 & 0 & 0 & 0 & 0 & 0 & 0 & 0\\ k_{5,1} & k_{5,2} & k_{5,3} & 0 & 0 & 0 & 0 & 0 & 0 & 0 & 0\\ k_{6,1} & k_{6,2} & k_{6,3} & 0 & 0 & 0 & 0 & 0 & 0 & 0 & 0\\ k_{7,1} & k_{7,2} & k_{7,3} & 0 & 0 & 0 & 0 & 0 & 0 & 0 & 0\\ k_{8,1} & k_{8,2} & k_{8,3} & 0 & 0 & 0 & 0 & 0 & 0 & 0 & 0\\ k_{9,1} & k_{9,2} & k_{9,3} & 0 & 0 & 0 & 0 & 0 & 0 & 0\\ k_{10,1} & k_{10,2} & k_{10,3} & 0 & 0 & 0 & 0 & 0 & 0 & 0 & 0\\ k_{11,1} & k_{11,2} & k_{11,3} & 0 & 0 & 0 & 0 & 0 & 0 & 0 & 0 \end{array}\right]$$

where $E_{0}=\left(D_{P}^{s*},\;D_{U}^{s*},\;D_{R}^{s*},0,0,0,\;D_{P}^{T*},\;D_{U}^{T*},\;D_{R}^{T*},\;O_{P}^{s*},\;O_{R}^{s*},0,0,\;O_{P}^{v*},\;O_{R}^{v*},\;J_{P}^{s*},\;J_{U}^{s*},\;J_{R}^{s*},0,0,0,0,0,0,0,0,0,\;A_{P}^{s*},\;A_{U}^{s*},\;A_{R}^{s*},0,0,0,0,0,0,0,0,0,0,0,0\right),$ with $D_{P}^{s*}=\frac{\mu_{D}N_{P}^{D}\left(d_{D}+\tau_{T}\right)}{d_{D}\left(d_{D}+\gamma_{T}^{D}+\tau_{T}\right)},D_{U}^{s*}=\frac{\mu_{D}N_{U}^{D}\left(d_{D}+\tau_{T}\right)}{d_{D}\left(d_{D}+\gamma_{T}^{D}+\tau_{T}\right)},D_{R}^{s*}=\frac{\mu_{D}N_{R}^{D}\left(d_{D}+\tau_{T}\right)}{d_{D}\left(d_{D}+\gamma_{T}^{D}+\tau_{T}\right)},\;D_{P}^{T*}=\frac{\mu_{D}N_{P}^{D}\gamma_{T}^{D}}{d_{D}\left(d_{D}+\gamma_{T}^{D}+\tau_{T}\right)},\;D_{U}^{T*}=\frac{\mu_{D}N_{U}^{D}\gamma_{T}^{D}}{d_{D}\left(d_{D}+\gamma_{T}^{D}+\tau_{T}\right)},D_{R}^{T*}=\frac{\mu_{D}N_{R}^{D}\gamma_{T}^{D}}{d_{D}\left(d_{D}+\gamma_{T}^{D}+\tau_{T}\right)},\;O_{P}^{s*}=\frac{\mu_{O}N_{P}^{O}\left(d_{0}+\gamma_{sv}^{O}\right)}{d_{O}\left(d_{O}+v+\gamma_{sv}^{O}\right)},\;O_{R}^{s*}=\frac{\mu_{O}N_{R}^{O}\left(d_{0}+\gamma_{sv}^{O}\right)}{d_{O}\left(d_{O}+v+\gamma_{sv}^{O}\right)},\;O_{P}^{v*}=\frac{\mu_{O}N_{P}^{O}v}{d_{O}\left(d_{O}+v+\gamma_{sv}^{O}\right)},O_{R}^{v*}=\frac{\mu_{O}N_{R}^{O}v}{d_{O}\left(d_{O}+v+\gamma_{sv}^{O}\right)},\;J_{P}^{s*}=\frac{\mu_{H}N_{P}^{H}}{d_{H}+\tau },\;J_{U}^{s*}=\frac{\mu_{H}N_{U}^{H}}{d_{H}+\tau },\;J_{R}^{s*}=\frac{\mu_{H}N_{R}^{H}}{d_{H}+\tau },\;A_{P}^{s*}=\frac{\mu_{H}N_{P}^{H}\tau }{d_{H}\left(d_{H}+\tau \right)},A_{U}^{s*}=\frac{\mu_{H}N_{U}^{H}\tau }{d_{H}\left(d_{H}+\tau \right)}$, and $A_{R}^{s*}=\frac{\mu_{H}N_{R}^{H}\tau }{d_{H}\left(d_{H}+\tau \right)}$ is the disease-free equilibrium.

### Appendix A.3. The Target Reproductive Number

To calculate the target reproductive number of the control strategy aimed at reducing the transmission of the disease from sheep to dogs by vaccination, $\Gamma_{v},$ the following partition of the K,K=D+B, matrix was considered:

$$D=\left[\begin{array}{ccccccccccc} 0 & 0 & 0 & 0 & k_{1,5} & 0 & 0 & 0 & 0 & 0 & 0\\ 0 & 0 & 0 & 0 & 0 & 0 & 0 & 0 & 0 & 0 & 0\\ 0 & 0 & 0 & 0 & k_{3,5} & 0 & 0 & 0 & 0 & 0 & 0\\ 0 & 0 & 0 & 0 & 0 & 0 & 0 & 0 & 0 & 0 & 0\\ 0 & 0 & 0 & 0 & 0 & 0 & 0 & 0 & 0 & 0 & 0\\ 0 & 0 & 0 & 0 & 0 & 0 & 0 & 0 & 0 & 0 & 0\\ 0 & 0 & 0 & 0 & 0 & 0 & 0 & 0 & 0 & 0 & 0\\ 0 & 0 & 0 & 0 & 0 & 0 & 0 & 0 & 0 & 0 & 0\\ 0 & 0 & 0 & 0 & 0 & 0 & 0 & 0 & 0 & 0\\ 0 & 0 & 0 & 0 & 0 & 0 & 0 & 0 & 0 & 0 & 0\\ 0 & 0 & 0 & 0 & 0 & 0 & 0 & 0 & 0 & 0 & 0 \end{array}\right],$$

$$B=\left[\begin{array}{ccccccccccc} 0 & 0 & 0 & k_{1,4} & 0 & 0 & 0 & 0 & 0 & 0 & 0\\ 0 & 0 & 0 & 0 & 0 & 0 & 0 & 0 & 0 & 0 & 0\\ 0 & 0 & 0 & k_{3,4} & 0 & 0 & 0 & 0 & 0 & 0 & 0\\ k_{4,1} & k_{4,2} & k_{4,3} & 0 & 0 & 0 & 0 & 0 & 0 & 0 & 0\\ k_{5,1} & k_{5,2} & k_{5,3} & 0 & 0 & 0 & 0 & 0 & 0 & 0 & 0\\ k_{6,1} & k_{6,2} & k_{6,3} & 0 & 0 & 0 & 0 & 0 & 0 & 0 & 0\\ k_{7,1} & k_{7,2} & k_{7,3} & 0 & 0 & 0 & 0 & 0 & 0 & 0 & 0\\ k_{8,1} & k_{8,2} & k_{8,3} & 0 & 0 & 0 & 0 & 0 & 0 & 0 & 0\\ k_{9,1} & k_{9,2} & k_{9,3} & 0 & 0 & 0 & 0 & 0 & 0 & 0\\ k_{10,1} & k_{10,2} & k_{10,3} & 0 & 0 & 0 & 0 & 0 & 0 & 0 & 0\\ k_{11,1} & k_{11,2} & k_{11,3} & 0 & 0 & 0 & 0 & 0 & 0 & 0 & 0 \end{array}\right]$$

The target reproductive number for the set $S_{v}$ corresponds to the spectral radius of the matrix $D{\left(I-B\right)}^{-1},$ i.e., $\Gamma_{v}=\rho \left(D{\left(I-B\right)}^{-1}\right)$.

The target reproductive number of the control strategy designed to reduce disease transmission from dogs to sheep by implementing deworming measures considered the following *K*-matrix partition:

$$D=\left[\begin{array}{ccccccccccc} 0 & 0 & 0 & 0 & 0 & 0 & 0 & 0 & 0 & 0 & 0\\ 0 & 0 & 0 & 0 & 0 & 0 & 0 & 0 & 0 & 0 & 0\\ 0 & 0 & 0 & 0 & 0 & 0 & 0 & 0 & 0 & 0 & 0\\ k_{4,1} & 0 & 0 & 0 & 0 & 0 & 0 & 0 & 0 & 0 & 0\\ k_{5,1} & 0 & 0 & 0 & 0 & 0 & 0 & 0 & 0 & 0 & 0\\ \;k_{6,1} & 0 & 0 & 0 & 0 & 0 & 0 & 0 & 0 & 0 & 0\\ k_{7,1} & 0 & 0 & 0 & 0 & 0 & 0 & 0 & 0 & 0 & 0\\ k_{8,1} & 0 & 0 & 0 & 0 & 0 & 0 & 0 & 0 & 0 & 0\\ k_{9,1} & 0 & 0 & 0 & 0 & 0 & 0 & 0 & 0 & 0 & 0\\ k_{10,1} & 0 & 0 & 0 & 0 & 0 & 0 & 0 & 0 & 0 & 0\\ k_{11,1} & 0 & 0 & 0 & 0 & 0 & 0 & 0 & 0 & 0 & 0 \end{array}\right],$$

$$B=\left[\begin{array}{ccccccccccc} 0 & 0 & 0 & k_{1,4} & k_{1,5} & 0 & 0 & 0 & 0 & 0 & 0\\ 0 & 0 & 0 & 0 & 0 & 0 & 0 & 0 & 0 & 0 & 0\\ 0 & 0 & 0 & k_{3,4} & k_{3,5} & 0 & 0 & 0 & 0 & 0 & 0\\ 0 & k_{4,2} & k_{4,3} & 0 & 0 & 0 & 0 & 0 & 0 & 0 & 0\\ 0 & k_{5,2} & k_{5,3} & 0 & 0 & 0 & 0 & 0 & 0 & 0 & 0\\ 0 & k_{6,2} & k_{6,3} & 0 & 0 & 0 & 0 & 0 & 0 & 0 & 0\\ 0 & k_{7,2} & k_{7,3} & 0 & 0 & 0 & 0 & 0 & 0 & 0 & 0\\ 0 & k_{8,2} & k_{8,3} & 0 & 0 & 0 & 0 & 0 & 0 & 0 & 0\\ 0 & k_{9,2} & k_{9,3} & 0 & 0 & 0 & 0 & 0 & 0 & 0\\ 0 & k_{10,2} & k_{10,3} & 0 & 0 & 0 & 0 & 0 & 0 & 0 & 0\\ 0 & k_{11,2} & k_{11,3} & 0 & 0 & 0 & 0 & 0 & 0 & 0 & 0 \end{array}\right]$$

The target reproductive number for the set $S_{\gamma_{T}^{D}}$ corresponds to the spectral radius of the matrix $D{\left(I-B\right)}^{-1},$ i.e., $\Gamma_{\gamma_{T}^{D}}=\rho \left(D{\left(I-B\right)}^{-1}\right)$.
